# Nijmegen breakage syndrome fibroblasts expressing the C-terminal truncated NBN^p70^ protein undergo p38/MK2-dependent premature senescence

**DOI:** 10.1007/s10522-014-9530-3

**Published:** 2014-09-12

**Authors:** Terence Davis, Hannah S. E. Tivey, Amy J. C. Brook, David Kipling

**Affiliations:** Institute of Cancer and Genetics, School of Medicine, Cardiff University, Heath Park, Cardiff, CF14 4XN, UK

**Keywords:** Cellular morphology, Human ageing, MAPKAPK2, p38 MAP kinase inhibitors, Progeroid syndromes, Telomeres

## Abstract

Fibroblasts from the progeroid Nijmegen breakage syndrome that express a truncated version of the nibrin protein (NBN^p70^) undergo premature senescence and have an enlarged morphology with high levels of senescence-associated β-galactosidase, although they do not have F-actin stress fibres. Growth of these fibroblasts in the continuous presence of p38 inhibitors resulted in a large increase in replicative capacity and changed the cellular morphology so that the cells resembled young normal fibroblasts. A similar effect was seen using an inhibitor of the p38 downstream effector kinase MK2. These data suggest that NBN^p70^ expressing cells undergo a degree of stress-induced replicative senescence via p38/MK2 activation, potentially due to increased telomere dysfunction, that may play a role in the progeroid features seen in this syndrome.

**Electronic supplementary material:**

The online version of this article (doi:10.1007/s10522-014-9530-3) contains supplementary material, which is available to authorized users.

## Introduction

Nijmegen Breakage Syndrome (NBS) is an autosomal recessive disorder characterised by microcephaly, growth retardation, and immunodeficiency accompanied by recurrent infection and lymphoma (Ochs et al. 1997). NBS has been described as a progeroid (rapid ageing) syndrome (Hofer et al. 2005). NBS individuals display progeroid-like features, such as sparse and grey hair, café-au-lait spots and distinctive ‘bird-like’ facies, characterised by a receding forehead, a prominent mid-face with a long nose, and a receding mandible, all of which become more pronounced with age (Seemanova et al. 1985). Human ageing has been linked to the build up of senescent cells during life since they display deleterious biochemical features resulting from the expression of degradative enzymes and inflammatory cytokines, and may contribute to age-related degenerations in division-competent tissues as a result of reduced proliferative capacity in organs where cell division is central to normal function or repair (Kipling et al. 2004; Burton 2009). Primary lymphocytes and fibroblasts recently explanted from NBS individuals typically show slow growth and undergo premature replicative senescence that, if recapitulated in vivo, may underlie some of the progeroid features seen in NBS (Ranganathan et al. 2001; Cabuy et al. 2005).

Progeroid syndromes (PSs) are widely used as models for human ageing because, in those aspects where premature ageing occurs, the process and pathology are similar to that seen in normally aged individuals (Davis et al. 2009; Hofer et al. 2005). The best-studied PSs are Werner Syndrome (WS), Ataxia Telangiectasia (AT) and Hutchinson Gilford Progeria (HGPS), which carry causal mutations in the WRN RecQ helicase, ataxia telangiectasia checkpoint kinase ATM, and lamin A/C respectively (Hofer et al. 2005). Fibroblasts from these individuals show accelerated ageing in vitro, with WS and AT fibroblasts having a very abbreviated replicative capacity and HGPS fibroblasts showing high levels of apoptosis (Tollefsbol and Cohen 1984; Tchirkov and Lansdorp 2003; Bridger and Kill 2004). Based upon such studies it has been postulated that premature cellular senescence may underlie many of the ageing features seen in PSs (Faragher et al. 2009). However, recent data has shown that premature cellular senescence only occurs in a subset of PSs that includes WS, AT, HGPS, and ATR-SS (a version of Seckel Syndrome that has mutations in the AT and Rad3-related checkpoint kinase ATR) (Tivey et al. 2013a, 2013b; Davis et al. 2013b).

Cellular senescence in many human cell types results from the inability of DNA replication to maintain the telomeres at the ends of chromosomes resulting in telomere erosion during cell division. Eventually, the telomeres shorten to the extent that cell division is halted and the cells enter replicative senescence (Allsopp et al. 1992). Nibrin (NBN), the protein affected in NBS, has been suggested to play a role in telomere length maintenance (Ranganathan et al. 2001; Verdun and Karlseder 2006). However, although newly-isolated NBS fibroblasts have relatively short telomeres when compared to newly-isolated normal fibroblasts, there does not appear to be a significantly increased rate of telomere erosion *per se,* with the replicative capacities of NBS cells being inversely proportional to the telomere length at explant (Tauchi et al. 2002; Hou et al. 2012). This does not exclude a telomere defect in NBS cells, however, and there is evidence that the numbers of telomere fusions are elevated (Zhang et al. 2005; Cabuy et al. 2005). In addition, ectopic cellular expression of an NBN mutant protein that cannot be activated by ATM results in telomere truncations and fusions (Bai and Murnane 2003), as does RNAi knockdown of NBN (Zhang et al. 2005). These data suggest that the replicative cellular defect may be due to an increased incidence of telomere fusions, although the mechanism is not well understood (Ranganathan et al. 2001).

Like with NBS, there appears to be no increased rate of telomere erosion in WS or ATR-SS fibroblasts (Baird et al. 2004; McNees et al. 2010), suggesting their premature senescence is independent of telomere erosion. However, both WS and ATR-SS fibroblasts show telomere instability (Ariyoshi et al. 2007; Crabbe et al. 2004; Pirzio et al. 2008; Casper et al. 2004, 2002), which may explain the observed increased frequency of telomere-derived sister chromatin exchanges (T-SCEs) seen in these syndromes (McNees et al. 2010; Hagelstrom et al. 2010). The premature senescence seen in WS and ATR-SS fibroblasts appears due, at least in part, to the activation of the stress-associated p38 MAPK resulting in what has been termed SIPS (stress-induced premature senescence) (Toussaint et al. 2002a). p38 inhibition is able to extend the replicative capacity of WS and ATR-SS fibroblasts considerably (Davis et al. 2005; Tivey et al. 2013b). Because telomere instability is known to activate p38 (Iwasa et al. 2003), these data suggest that some of the premature senescence phenotype may be due to SIPS resulting from increased telomere instability.

As NBS has progeroid features, accelerated fibroblast senescence, and telomere fusions, and NBN is involved in many of the same DNA damage response pathways as both WRN and ATR (Pichierri and Franchitto 2004; Shiotani et al. 2013), we hypothesised that premature senescence in NBS cells might result from activation of p38 signalling and SIPS. To address this we used a range of inhibitors of the p38-signalling pathway, together with molecular profiling, to test whether the accelerated senescence seen in NBS fibroblasts requires the p38-signalling pathway.

## Materials and methods

The primary dermal fibroblasts used in this work were derived from biopsies of human tissue and obtained from the Coriell Cell Repository (Camden, NJ, USA). GM07166 is from a 20 year old female homozygous for a deletion of 5 nucleotides in exon 6 of the *NBS1* gene resulting in a frameshift, and premature termination at codon 218. Immunoblot confirms the absence of wild-type NBN protein in this strain and the presence of low levels of a C-terminal peptide termed NBN^p70^ (Yanagihara et al. 2011); these are referred to as NBS^p70^ cells throughout this manuscript. The normal dermal fibroblast strains (NDFs) have been described previously (Tivey et al. 2013a).

All cells were grown in Dulbecco’s modified Eagle medium (DMEM: Gibco Invitrogen, Paisley, UK) supplemented with 10 % foetal calf serum (Source BioScience UK Limited, Nottingham), 2 mM l-glutamine (Invitrogen Life Technologies Ltd., Paisley, UK), 10,000 U/ml penicillin and 10 mg/ml streptomycin (Sigma, Poole, UK) in an atmosphere of 21 % O_2_ and 5 % CO_2_, and passaged every 4–5 days as described previously (Davis et al. 2003). Cultures were not allowed to become confluent at any time in order to maintain maximal growth rates. Population doublings (PDs) were calculated according to the formula: PDs = log(*N*_t_/*N*_o_)/log2, where *N*_t_ is number of cells counted and *N*_o_ is number of cells seeded.

For experiments using kinase inhibitors the medium was supplemented with 2.5 µM SB203580 (Tocris Chemical Co. Bristol, UK), 2.5 µM BIRB 796 (Bagley et al. 2006), 0.5 µM VX-745 (Bagley et al. 2007) or 5 µM MK2.III [Merck, UK (Anderson et al. 2007)] dissolved in DMSO, with the medium changed daily. For controls, an equivalent volume of DMSO was added to the medium.

To activate the p38-signalling pathway using anisomycin, immortalised normal AG16409 cells (NDF^tert^) were plated onto 100 mm dishes in DMEM and cultured for 2 days at 37 °C, after which the cells were treated with 30 µM anisomycin (Sigma, Poole, UK) for 45 min (Davis et al. 2013a).

All other materials and methods used in this article including immunoblotting, antibodies, senescence-associated β-galactosidase assay (SAβ-gal), and FITC-phalloidin immunofluorescence have been described previously (Davis et al. 2013b).

## Results

### NBS^p70^ fibroblasts undergo premature cellular senescence

**Fig. 1:**
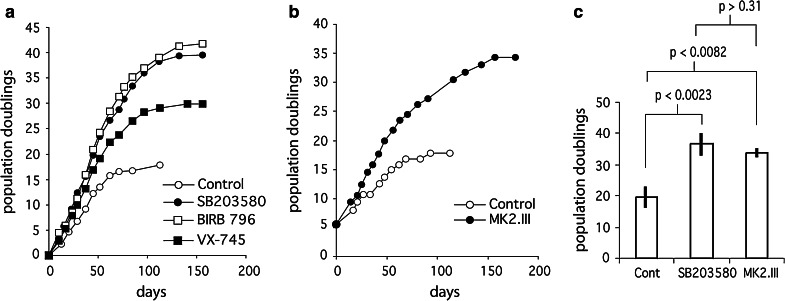
Growth characteristics of NBS cells. **a** example growth curves in PDs versus days for primary NBS^p70^ cells supplemented with 0.1 % (v/v) DMSO (*open circle*), 2.5 µM SB203580 (*fill circle*), 2.5 µM BIRB 796 (*open square*) or 0.5 µM VX-745 (*fill square*). **b** example growth curves in PDs versus days for primary NBS^p70^ cells supplemented with 0.1 % (v/v) DMSO (*open circle*) or 5.0 µM MK2.III (*fill circle*): note that the NBS^p70^ cells had already achieved 5.6 PDs prior to starting the growth experiment. **c** comparison of the mean replicative capacity for control NBS^p70^ cells and NBS^p70^ cells treated with SB203580 or MK2.III (Student’s *t*-test)

Primary NBS^p70^ fibroblasts were grown to senescence in DMEM either with no supplementation, or in medium that had been supplemented with various p38 or MK2 inhibitors (illustrated in Fig. 1a, b). Control NBS^p70^ fibroblasts had a replicative capacity of 19.6 ± 3.0 PDs (Table 1) that was statistically different (*p* < 0.036) from the mean replicative capacity of eight NDF strains of 38.8 ± 10.5 PDs (Table S1). The NBS^p70^ replicative capacity was less than that of all the normal fibroblasts strains used.

**Table 1:** Lifespan of NBS^p70^ fibroblasts

Condition	PDs achieved^a^	*Probability* ^b^
Control	19.6 ± 3.0 (n = 3)	na
VX-745	30.1 (n = 1)	*p* < 0.005^c^
SB203580	36.5 ± 3.0 (n = 3)	*p* < 0.002^d^
BIRB 796	41.7 (n = 1)	*p* < 0.0001^c^
MK2.III	33.8 ± 0.8 (n = 2)	*p* < 0.0082^d^

^a^Mean ± SD (sample number) except when a single sample has been analyzed

^b^Probability that the lifespan is same as for untreated cells

^c^ *z*-test

^d^Student’s *t*-test

### Inhibition of p38 rescues the premature senescence phenotype of NBS^p70^ cells

Treatment with SB203580 at 2.5 µM increased the NBS^p70^ replicative capacity to 36.5 ± 3.0 PDs, a highly statistically significant increase compared to untreated cells (Table 1; Fig. 1c). The resulting NBS^p70^ replicative capacity was now within the range of the SB203580-treated NDFs of 46.6 ± 12.1 PDs (*p* > 0.21) (Table S1); indeed it was greater than two of the previously used normal strains. Additionally the percentage lifespan increase achieved using SB203580 on NBS^p70^ cells of approximately 90 % was considerably greater than previously seen with NDFs of approximately 30 % (Tivey et al. 2013a). Significantly increased replicative capacities were also seen when NBS^p70^ cells were treated with the p38 inhibitors BIRB 796 (at 2.5 µM) and VX-745 (at 0.5 µM) (Table 1), which again brought the NBS^p70^ replicative capacity to within the range seen for inhibitor-treated NDFs (Table S2). The p38 inhibitors also resulted in an increased growth rate for the NBS^p70^ cells. Finally, treatment of NBS^p70^ cells with the MK2 inhibitor MK2.III (Anderson et al. 2007) significantly increased the replicative capacity to 33.8 ± 0.8 PDs (Table 1), an increase in experimental lifespan of 101 % (taking into account that the NBS^p70^ cells were at 5.6 PDs when MK2 inhibition began; Fig. 1b) which was much greater than has been seen using this inhibitor on NDFs (Davis et al. 2013a). Additionally, MK2 inhibition resulted in a more rapid growth rate in the NBS^p70^ cells (Fig. 1b).

### NBS^p70^ fibroblasts do not show elevated levels of F-actin stress fibres

**Fig. 2:**
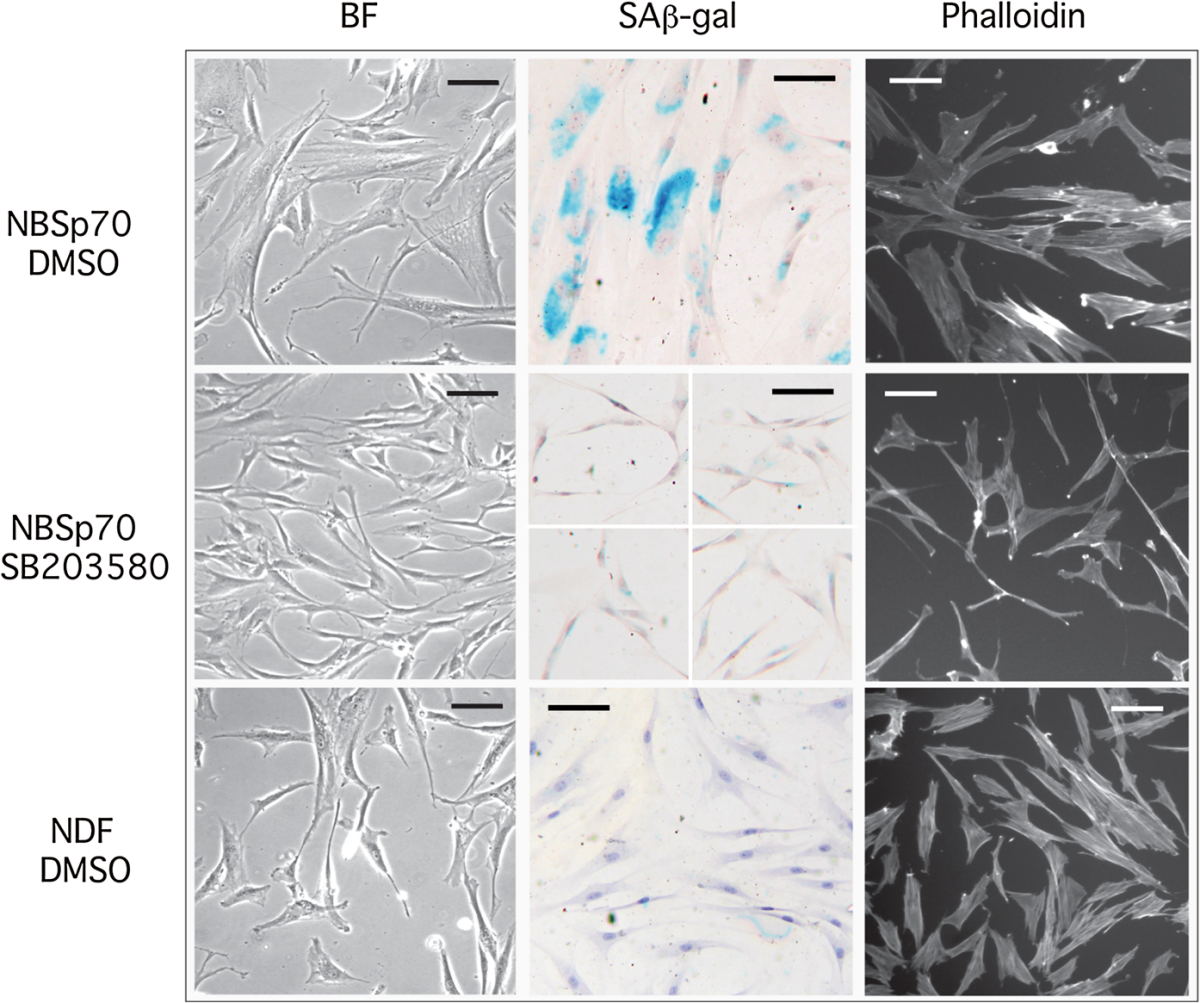
Cellular morphology, SAβ-gal staining and phalloidin-FITC staining: *BF* brightfield; *bar* = 100 µm. The *panel* for GM07166 SB203580 SAβ-gal is a composite of four separate views due to the *low cell* density; however, the *scale* is the same as for the other *panels*

In addition to a reduced replicative capacity, fibroblasts isolated from WS and ATR-SS individuals had an enlarged morphology with extensive F-actin stress fibres that resembled senescent cells (Davis et al. 2005; Tivey et al. 2013b). Stress fibres result from activation of p38 via its downstream target MK2 that phosphorylates the actin binding protein HSP27 (Shi et al. 2003). Many low PD NBS^p70^ cells had an enlarged morphology with an aged and granular appearance that resembled senescent cells when compared to low PD AG16409 NDFs, and had an SAβ-gal staining index of 37.6 %. However, in contrast to WS cells the NBS^p70^ cells did not have F-actin stress fibres (Fig. 2, top panels). Following SB203580 treatment NBS^p70^ cells were reduced in size, had an SAβ-gal index of 3 % (Fig. 2, middle panels), and now resembled control AG16409 NDFs (Fig. 2, bottom panels). Similar results were seen with BIRB 796, VX-745 and MK2.III (not shown).

### Levels of p38 pathway activation are similar in NBS^p70^ and normal cells

**Fig. 3:**
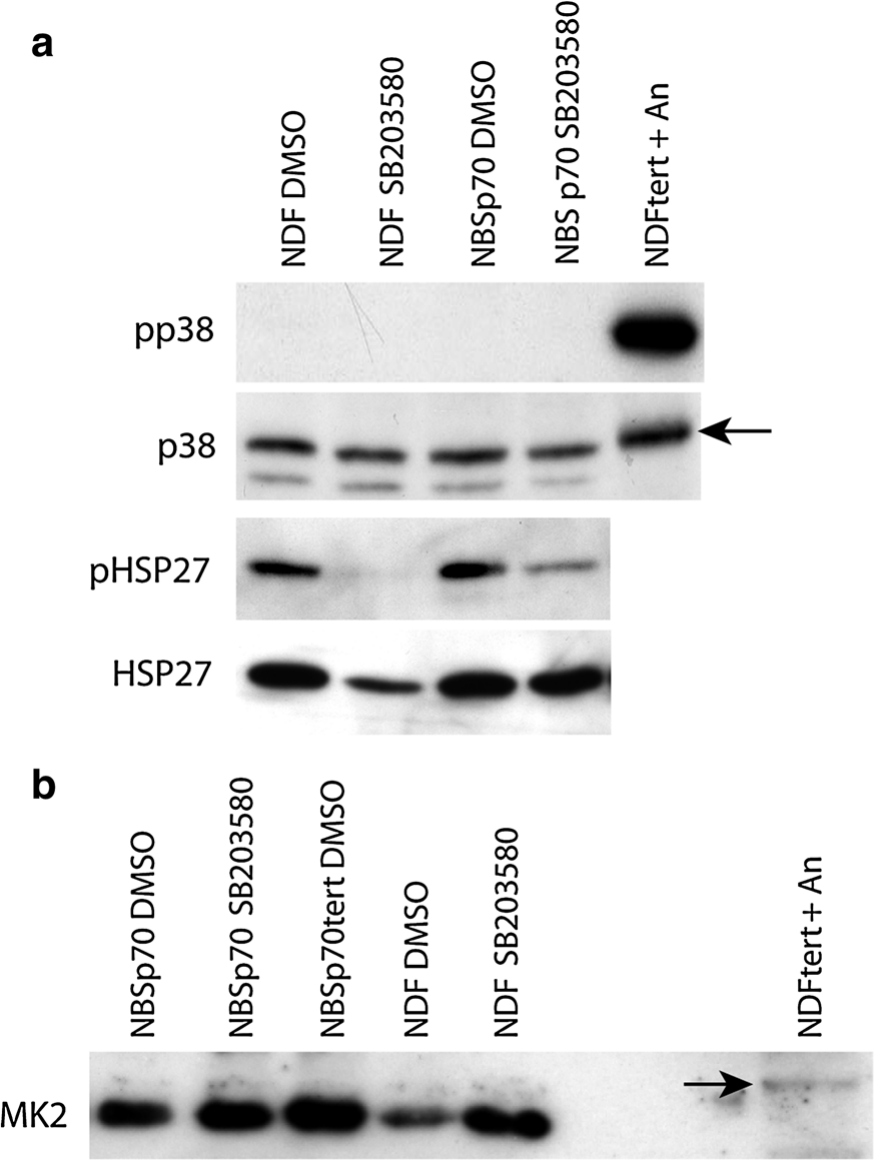
Analysis of stress kinases in NBS^p70^ cells and NDFs. **a** lysates were prepared from cells grown in DMEM + 0.1 % (v/v) DMSO **d** or 2.5 µM SB203580 (SB). Proteins are p38 (*arrow* indicates p38 band), phospho-p38 (pp38), HSP27 and phospho-HSP27 (pHSP27). NDFtert + An are telomerase immortalised NDFs treated with 30 µM anisomycin for 45 min to activate p38. **b** lysates were prepared as for (**a**). Protein is MK2, *arrow* indicates phosphorylated MK2 (activated) in the control lane. NBSp70tert are telomerase immortalised NBS^p70^ cells used as a control

Fibroblasts from both WS and ATR-SS individuals show high levels of activated p38 and phosphorylated HSP27 that correlate with their reduced replicative capacity and extensive stress fibres (Davis et al. 2005; Tivey et al. 2013b). Thus, protein extracts were prepared from low PD primary NBS^p70^ cells and immunoblotted with antibodies specific to the phosphorylated forms of p38 and HSP27 to assess activation of the p38 signalling pathway. Phosphorylated (activated) p38 was not detected in either NBS^p70^ cells or in the NDF strain AG16409 (Fig. 3a). Similarly, although some detectable phosphorylated HSP27 (a p38 downstream target) was found in NBS^p70^ cells, this was at levels similar to that seen in NDFs. The level of phosphorylated HSP27 was reduced with SB203580 treatment in both NBS^p70^ cells and NDFs. The low levels of phosphorylated HSP27 correlate with the lack of F-actin stress fibres seen in NBS^p70^ cells. Likewise no activated MK2 was seen in NBS^p70^ cells or NDFs, as assessed by the lack of a band shift (see control lane in Fig. 3b) when using an MK2 antibody that recognises both active and inactive MK2.

## Discussion

Normal human fibroblasts have a limited division potential and undergo telomere-driven replicative senescence (Herbig et al. 2004; Allsopp and Harley 1995). At senescence the fibroblasts have an enlarged granular appearance with extensive arrays of F-actin stress fibres (Davis et al. 2005). Short telomeres activate the tumour suppressor p53 that then up-regulates p21^WAF1^ resulting in cell cycle arrest. In addition to activation of p53, short telomeres activate the stress kinase p38 that can also induce cell cycle arrest (Iwasa et al. 2003). Activation of p38 results in the production of F-actin stress fibres via MK2 and HSP27 phosphorylation (Shi et al. 2003). Thus the features seen in seen in replicatively senescent cells result from activation of two distinct molecular pathways (Iwasa et al. 2003). In addition to telomere-induced replicative senescence, fibroblasts can undergo a stress-induced cell cycle arrest as a result of activation of p38 leading to what is termed stress-induced premature senescence or SIPS (Toussaint et al. 2002b). SIPS can occur through both exogenous stress such as high oxygen levels, or endogenous stress as a result of difficulties during DNA replication or repair (Toussaint et al. 2002b). These two processes of telomere-dependent senescence and SIPS synergise to determine the replicative capacity of human fibroblasts (Tivey et al. 2013a).

Fibroblasts derived from various progeroid syndromes have been shown to have a reduced replicative capacity compared to normal cells; such syndromes include WS, ATR-SS, AT and HGPS (Davis and Kipling 2009; Tivey et al. 2013a; Davis et al. 2005; Tivey et al. 2013b). This reduced replicative potential has been suggested as contributing to the accelerated ageing seen in these syndromes (Kipling et al. 2004). In some cases the reduced replicative capacity is due to increased telomere dysfunction (AT), and in others due to problems with DNA replication causing replication stress that lead to high activation of p38 and a high degree of SIPS (ATR-SS and WS).

NBS is considered a progeroid syndrome and NBS^p70^ cells have a much-reduced replicative capacity compared to NDFs. Continuous treatment of NBS^p70^ fibroblasts with several p38 inhibitors resulted in large significant increases in replicative capacity to within the range seen in inhibitor treated NDFs. The increases seen for NBS^p70^ cells were also significantly greater than the increases seen for inhibitor treated NDFs. The inhibitors SB203580 and BIRB 796 are more effective at extending replicative capacity than VX-745 that is probably due to their greater ability to inhibit p38 continuously throughout the long-term growth of the cells, as has been seen previously using ATR-SS cells (Tivey et al. 2013b). However, although NBS^p70^ fibroblasts have an enlarged morphology, high SAβ-gal staining levels and resemble aged cells to some degree, all features that are corrected with p38/MK2 inhibition, they do not have F-actin stress fibres and there is no obvious activation of p38 or MK2. As the p38 inhibitors are of different chemotypes and have different modes of action and potencies (Bagley et al. 2010), and the effects of p38 inhibitors in NBS^p70^ cells are matched by an MK2 inhibitor that is a known downstream effector of p38 (Kyriakis and Avruch 2001), it seems unlikely that these results are due to a target other than the p38-signalling pathway. These data suggest that the premature senescence seen in NBS^p70^ primary dermal fibroblasts is due (at least in part) to p38 activation primarily through activation of the kinase MK2. However, further work using siRNA technology to target MK2 should be done to corroborate these results when suitable cell material becomes available; siRNA to target p38 cannot be done for long-term growth experiments as p38 knockdown results in cell lethality (our unpublished data).

Given the effect of p38 inhibition on NBS^p70^ cells, why is no expression of activated p38 seen in these cells? A similar situation is seen in NDFs and in fibroblasts from several other PSs, where p38 inhibition similarly extends cellular replicative capacity despite activated p38 not being detectable by immunoblot (Tivey et al. 2013a; Davis and Kipling 2009). It may be that p38 is activated in a transient, cell cycle dependent, fashion; and indeed a functional role has been suggested for p38 in regulating the G2/M transition in the absence of stress, with p38 activation only being transiently detected at the G2/M transition (Cha et al. 2007). Another example come from the use of nucleoside analogs to slow the rate of DNA replication in U2OS cells (Kopper et al. 2013). Such treatment leads to a growth arrest that can be prevented by MK2 inhibition using MK2.III, yet no MK2 activation was seen by immunoblot; is has been speculated that a low level of MK2 activation occurs that is confined to the cell nucleus in the nucleoside treated cells (Kopper et al. 2013). Whatever their mechanism of action, both serve to illustrate that p38 or MK2 can be activated sufficiently to have an effect upon the cell cycle and cellular replicative capacity, but not sufficiently to make this activation readily detectable by immunoblot, and thus by extrapolation potentially not sufficiently to affect cellular morphology by triggering the expression of F-actin stress fibres. Therefore it is possible that the defect in NBS^p70^ cells activates p38/MK2 at a specific time in the cell cycle at a level that is not detectable using immunoblot, but that is sufficient to cause a significant level of cell cycle arrest and the observed reduced replicative capacity.

The effect of p38 inhibition on replicative capacity in NBS^p70^ cells is similar to that seen in ATR-SS cells, but smaller than seen in WS cells. Most of the increase in replicative capacity in NBS^p70^ cells resulted from MK2 inhibition, whereas MK2 inhibition only resulted in a moderate increase in replicative capacity in WS cells when compared to p38 inhibition, and MK2 inhibition had little effect in NDFs (Davis et al. 2013a). In addition, MK2 inhibition increased the growth rates of NBS^p70^ cells, but had little effect in either WS cells or NDFs. Finally, both ATR-SS and WS cells show extensive F-actin stress fibres and p38 activation that are not seen in NBS^p70^ cells (Tivey et al. 2013a; Davis et al. 2005; Tivey et al. 2013b). Thus, the NBS^p70^ cell phenotypes and their responses to kinase inhibition appear to be intermediate between that of WS and ATR-SS cells, and NDFs (summarised in Table S3).

In WS or ATR-SS cells the lack (or low levels) of WRNp and ATR results in a high level of replication stress (Ammazzalorso et al. 2010; McNees et al. 2010; Ozeri-Galai et al. 2008; Pirzio et al. 2008) due to a high degree of stalling at DNA replication forks (Rodriguez-Lopez et al. 2002; Koundrioukoff et al. 2013), particularly at hard-to-replicate sequences such as common fragile sites (CFS), and these sites are expressed in both WS and ATR-SS cells (Casper et al. 2004; Pirzio et al. 2008). In contrast, replication fork stalling and CFS expression have not been reported for NBS^p70^ expressing cells. In addition, both WS and ATR-SS syndrome cells have an increased level of telomere dysfunction (Ariyoshi et al. 2007; Crabbe et al. 2004; Pirzio et al. 2008; Casper et al. 2004, 2002; McNees et al. 2010), and an increase in telomere fusions is seen in NBS^p70^ cells that is also indicative of telomere dysfunction (Zhang et al. 2005; Cabuy et al. 2005). As both telomere dysfunction and replication stress can activate p38 (Iwasa et al. 2003; Llopis et al. 2012), the data suggest that the differences in the fibroblast behaviour from the three syndromes may simply relate to the nature of the stress occurring. It is possible that p38 is activated to a greater extent in WS and ATR-SS than is seen in NBS as two p38-activating processes are occurring, replication stress and telomere dysfunction. In NBS cells the only p38-acitvating process appears to be telomere dysfunction. Thus, the defect seen in NBS^p70^ cells results in a lower level of stress than defects seen in WS or ATR-SS, and subsequently a lower level of p38 activation.

The data in this paper also suggest that telomere dysfunction may signal through MK2 since its inhibition is almost as effective at extending replicative capacity in NBS^p70^ cells as p38 inhibition, which is a suggestion that appears to be novel to this work. Further support for this idea comes from the use of telomerase (tert) immortalised NBS^p70^ cells. Ectopic expression of tert in these cells does not result in telomere elongation (unlike in tert-immortalised NDFs) but merely maintains short telomere lengths, and the telomere fusions seen in primary NBS^p70^ cells are still prevalent, suggesting that NBN^p70^ has a dominant negative effect on aspects of telomere maintenance (Ranganathan et al. 2001; Siwicki et al. 2003). Tert-immortalised NBS^p70^ fibroblasts have a very slow growth rate compared to tert-immortalised NDFs (0.16 ± 0.005 PDs/day compared to 0.49 ± 0.02 PDs/day), and MK2 inhibition has a significant positive effect on this growth rate (0.23 ± 0.01 PDs/day; *p* < 0.0006), although it does not approach that of NDFs. In contrast, MK2 inhibition has no effect on the growth rate of tert-immortalised NDFs that have little (or no) telomere dysfunction (0.47 ± 0.02 PDs/day; *p* > 0.25). Overall, the data presented in this paper suggest that NBS^p70^ fibroblasts undergo a form of premature senescence, possibly as a result of difficulties in the replication of telomeres, and this is transduced via the p38/MK2-signalling pathway. Further work on the possible link between telomere dysfunction and MK2 activity is clearly warranted in other systems.

The differential activation of p38 in fibroblasts from the different PSs may correlate with, or indeed influence, the in vivo progeroid phenotypes seen, as the progeroid features are less pronounced in NBS compared to either WS or ATR-SS. In addition, a feature of both WS and ATR-SS is the presence of inflammatory conditions such as type II diabetes (Rauch 2011; Martin et al. 1999), which is in contrast to NBS where inflammatory conditions are not found (Hofer et al. 2005); activated p38 is strongly associated with inflammatory conditions (Kumar et al. 2003). It is also possible, if activation of p38/MK2 and premature cellular senescence do play a role in the aged features of these syndromes, that p38 and/or MK2 inhibitors may be a therapeutic possibility (Davis and Kipling 2006).

## Electronic supplementary material

Below is the link to the electronic supplementary material.

Supplementary material 1 (DOC 36 kb)

Supplementary material 2 (DOC 30 kb)

Supplementary material 3 (DOC 36 kb)
