# Supplementary material for: Nijmegen breakage syndrome fibroblasts expressing the C-terminal truncated NBNp70 protein undergo p38/MK2-dependent premature senescence
Source: Biogerontology. 2014 Sep 12;16(1):43–51. doi: 10.1007/s10522-014-9530-3 (PMC4305097; doi:10.1007/s10522-014-9530-3)
Supplement: Supplementary file 1 — Supplementary material 1 (DOC 36 kb) [file 10522_2014_9530_MOESM1_ESM.doc]

**Nijmegen Breakage syndrome fibroblasts expressing the C-terminal truncated NBN^p70^ protein undergo p38/MK2-dependent premature senescence**

Terence Davis • Hannah S. E. Tivey • Amy J. C. Brook • David Kipling

Cardiff University

davist2@cardiff.ac.uk

**Table S1. Fibroblast replicative capacity**

Strain ^a^ PDs achieved ^b^ PDs achieved ^b^

(control) (SB-treated)

*NDFs*

AG04552 ^c^ 24.3 30.1

AG06234 ^d^ 34.1 41.6

AG09603 ^c^ 47.8 55.7

AG11020 ^c^ 41.7 46.5

AG11081 ^d^ 33.4 39.3

AG13152 ^c^ 28.0 35.6

AG13156 ^d^ 46.6 63.4

AG16409 ^d^ 54.3 60.5

*Mean 38.8 ± 10.5 46.6 ± 12.1*

*NBS*

GM07166 19.6 ± 3.0 36.5 ± 3.0

*Probability* ^e^ *p < 0.036 p > 0.21*

^a^ All from Coriell Cell Repositories

^b^ Cells grown in EMEM

^c^ Data adapted from (Davis & Kipling 2009)

^d^ Data adapted from (Tivey et al. 2013a)

^e^ Probability that result for NBS cells is the same as seen for the NDFs; *z*-test.

Davis T, Kipling D (2009) Assessing the role of stress signalling via p38 MAP kinase in the premature senescence of ataxia telangiectasia and Werner syndrome fibroblasts. Biogerontology 10:253-266

Tivey HS, Brook AJ, Rokicki MJ, Kipling D, Davis T (2013) p38 (MAPK) stress signalling in replicative senescence in fibroblasts from progeroid and genomic instability syndromes. Biogerontology 14:47-62
