# Supplementary material for: Nijmegen breakage syndrome fibroblasts expressing the C-terminal truncated NBNp70 protein undergo p38/MK2-dependent premature senescence
Source: Biogerontology. 2014 Sep 12;16(1):43–51. doi: 10.1007/s10522-014-9530-3 (PMC4305097; doi:10.1007/s10522-014-9530-3)
Supplement: Supplementary file 2 — Supplementary material 2 (DOC 30 kb) [file 10522_2014_9530_MOESM2_ESM.doc]

**Nijmegen Breakage syndrome fibroblasts expressing the C-terminal truncated NBN^p70^ protein undergo p38/MK2-dependent premature senescence**

Terence Davis • Hannah S. E. Tivey • Amy J. C. Brook • David Kipling

Cardiff University

davist2@cardiff.ac.uk

**Table: S2 Lifespan of fibroblasts grown in presence of BIRB 796 and VX-745**

Strain PDs achieved

Cont. VX-745 BIRB 796

NDFs

AG06234 36.8 41.6 45.6

AG13152 28.0 31.7 38.3

AG16409 54.3 54.3 67.3

[*Mean ± SD 39.7 ± 13.3 42.5 ± 11.3 50.4 ± 15.1*](http://www.google.co.uk/imgres?q=T+TEST+formula&hl=en&client=firefox-a&sa=X&rls=org.mozilla:en-US:official&channel=np&biw=1513&bih=906&tbm=isch&prmd=imvns&tbnid=4dwtgr7EPOpPcM:&imgrefurl=http://imagesnoise.com/basic-statistics-formulas/17.htm&docid=5TuP-Zb4vJ1BpM&imgurl=http://imagesnoise.com/images/Basic%252520Statistics%252520Formulas/17.jpg&w=400&h=247&ei=_5DHT_jMFqKX0QWI6vmEDw&zoom=1&iact=hc&vpx=1188&vpy=173&dur=66&hovh=176&hovw=286&tx=154&ty=89&sig=118118992490773171377&page=1&tbnh=164&tbnw=261&start=0&ndsp=21&ved=1t:429,r:4,s:0,i:79)

NBS cells ^a^

GM07166 19.6 ± 3.0 (3) 30.0 (1) 41.7 (1)

*Probability* ^b^ *p > 0.13 p > 0.28*

^a^ NBS cultures were grown in triplicate for DMSO controls, and singly for VX-745 and BIRB 796.

^b^ Probability that this is same as for normal cells; *z*-test.
