# Supplementary material for: Nijmegen breakage syndrome fibroblasts expressing the C-terminal truncated NBNp70 protein undergo p38/MK2-dependent premature senescence
Source: Biogerontology. 2014 Sep 12;16(1):43–51. doi: 10.1007/s10522-014-9530-3 (PMC4305097; doi:10.1007/s10522-014-9530-3)
Supplement: Supplementary file 3 — Supplementary material 3 (DOC 36 kb) [file 10522_2014_9530_MOESM3_ESM.doc]

**Nijmegen Breakage syndrome fibroblasts expressing the C-terminal truncated NBN^p70^ protein undergo p38/MK2-dependent premature senescence**

Terence Davis • Hannah S. E. Tivey • Amy J. C. Brook • David Kipling

Cardiff University

davist2@cardiff.ac.uk

**Table S3 Comparison between normal, NBS^p70^, ATR-SS and WS fibroblasts**

**Condition** Normal ^a^ NBS^p70^ ATR-SS and WS ^a^

Replicative capacity 24-55 PDs 19 PDs 15-25 PDs

normal range reduced ^b^ reduced ^b^

effect of p38 inhibition increased lifespan, increased to within increased to within

30-63 PDs (SB203580) normal range ^c^ normal range ^c^

cell morphology normal, mostly small enlarged with high enlarged cells with

with few stress fibres SAβ-gal, no stress F-actin stress fibres

fibres

effect of SB203580 almost no effect cells resemble low PD cells resemble low PD

on morphology normal cells normal cells

phospho p38 level not detected not detected high

in low PD cells

effect of MK2 small ^d^ large (almost equal moderate for WS ^d^

inhibition to p38 inhibition) ^d^ not done for ATR-SS

^a^ Data adapted from (Davis et al. 2005; Tivey et al. 2013a; Tivey et al. 2013b)

^b^ Significantly different from normal

^c^ The increase for NBS^p70^ is similar to that seen for ATR-SS, but less than seen for WS cells

^d^ Data from (Davis et al. 2013a)

Davis T, Baird DM, Haughton MF, Jones CJ, Kipling D (2005) Prevention of Accelerated Cell Aging in Werner Syndrome Using a p38 Mitogen-Activated Protein Kinase Inhibitor. J Gerontol A Biol Sci Med Sci 60 (11):1386-1393

Davis T, Rokicki MJ, Bagley MC, Kipling D (2013a) The effect of small-molecule inhibition of MAPKAPK2 on cell ageing phenotypes of fibroblasts from human Werner syndrome. Chem Cent J 7 (1):18.

Tivey HS, Brook AJ, Rokicki MJ, Kipling D, Davis T (2013a) p38 (MAPK) stress signalling in replicative senescence in fibroblasts from progeroid and genomic instability syndromes. Biogerontology 14:47-62.

Tivey HS, Rokicki MJ, Barnacle JR, Rogers MJ, Bagley MC, Kipling D, Davis T (2013b) Small Molecule Inhibition of p38 MAP Kinase Extends the Replicative Life Span of Human ATR-Seckel Syndrome Fibroblasts. J Gerontol A Biol Sci Med Sci 68:1001-1009.
